# Five-Year Sustained Complete Remission With Minimal Adverse Effects Following Radiosurgery for 2-cm Brain Metastasis With Deep Eloquent Location From Lung Adenocarcinoma Despite Low Marginal Dose and High 12 Gy Volume

**DOI:** 10.7759/cureus.36680

**Published:** 2023-03-25

**Authors:** Kazuhiro Ohtakara, Makoto Nakao, Hideki Muramatsu, Kojiro Suzuki

**Affiliations:** 1 Department of Radiation Oncology, Kainan Hospital Aichi Prefectural Welfare Federation of Agricultural Cooperatives, Yatomi, JPN; 2 Department of Radiology, Aichi Medical University, Nagakute, JPN; 3 Department of Respiratory Medicine, Kainan Hospital Aichi Prefectural Welfare Federation of Agricultural Cooperatives, Yatomi, JPN

**Keywords:** radiotherapy treatment planning, single fraction stereotactic radiosurgery, volumetric modulated arc therapy, tyrosine kinase inhibitor, non-small cell lung cancer, lung adenocarcinoma, dose gradient, dose distribution, brain radionecrosis, brain metastasis

## Abstract

In single-fraction (sf) stereotactic radiosurgery (SRS) for brain metastases (BM) from lung adenocarcinoma (LAC), a marginal dose of ≥22-24 Gy is generally deemed desirable for achieving long-term local tumor control, whereas symptomatic brain radionecrosis significantly increases when the surrounding brain volume receiving ≥12 Gy (V_12 Gy_) exceeds >5-10 cm^3^, especially in a deep location. Here, we describe a 75-year-old male with a single LAC-BM of 20 mm in diameter, with a deep eloquent location, which was treated with sfSRS followed by erlotinib, resulting in sustained local complete remission (CR) with minimal adverse radiation effect at nearly five years after sfSRS. The LAC harbored epidermal growth factor receptor (EGFR) mutation. The gross tumor volume (GTV) was defined based on contrast-enhanced computed tomography (CECT) alone. sfSRS was implemented 11 days after planning CECT acquisition. The original GTV had some under- and over-coverage of the enhancing lesion. The D_98%_ values of corrected GTV (cGTV) (3.08 cm^3^) and 2-mm outside the cGTV were 18.0 Gy with 55% isodose and 14.8 Gy, respectively. The irradiated isodose volumes, including the GTV, receiving ≥22 Gy and ≥12 Gy were 2.18 cm^3^ and 14.32 cm^3^, respectively. Erlotinib was administered 13 days after sfSRS with subsequent dose adjustments over 22 months. There was a remarkable tumor response and subsequent nearly CR of the BM were observed at 2.7 and 6.3 months, respectively, with the tumor remnant being visible as a tiny cavitary lesion located in the cortex of the post-central gyrus at 56.4 months. The present case suggests the existence of: (i) extremely radio- and tyrosine kinase inhibitor (TKI)-sensitive LAC-BM for which sfSRS of ≤18 Gy combined with EGFR-TKI is sufficient for attaining long-term CR; and (ii) long-term brain tolerance following sfSRS despite high 12 Gy volume and deep eloquent location in the late 70s The moderate marginal dose of the GTV, the main location of the BM in the cerebral cortex, and the excellent tumor responses with sufficient extrication from the mass effect may render the BM immune to late adverse radiation effect.

## Introduction

Lung adenocarcinoma (LAC) is a common primary cancer for brain metastases (BMs). Stereotactic radiosurgery (SRS) is an important local treatment option, especially for limited BM cases [1]. In addition to the sophistication of the radiotherapy armamentarium, the ongoing progress of anti-cancer medications based on the integration of molecular diagnosis has enhanced systemic control and prognosis of LAC-BM cases [2,3]. According to the recently updated scores for estimating the prognosis of LAC-BM cases, i.e. Lung-molGPA (Graded Prognostic Assessment using molecular markers), the median survival time (MST) for LAC-BM patients with the favorable score of 3.5-4.0 is approximately four years [4]. Symptomatic BM as the first clinical manifestation of lung cancer, not otherwise specified, poses a therapeutic challenge in determining the optimal sequence of further evaluation and treatment [5,6]. In such clinical scenarios, long-term local tumor control (LTC) and safety are unprecedentedly expected for SRS, considering the potential long-term survival period [2,4,7,8].

Single-fraction (sf) SRS (sfSRS) is widely utilized for BM ≤3 cm in diameter from LAC and other primaries [1-3,7,9]. Generally, a prescription dose (PD) of 24 Gy is expected to provide one-year LTC probability of 95% for ≤20-mm BM, while 18 Gy is expected to provide one-year LTC probability of >85% for ≤20 mm and 75% for ≤30 mm [10]. A marginal dose of ≥22-24 Gy is deemed desirable for achieving long-term LTC with a high probability rate [9,10]. The PD is commonly assigned to the gross tumor volume (GTV) boundary under Leksell Gamma Knife® (LGK) (Elekta AB, Stockholm, Sweden), whereas various margin-added planning target volume (PTV) is usually the basis for the PD in the majority of linac-based SRS [9-11]. Additionally, the degrees of dose gradient outside and inside the isodose surface (IDS) for the PD along with the GTV dose heterogeneity substantially differ depending on modalities, irradiation techniques, and planning methods [7,11]. Consequently, D_98%_ of the GTV, which is a minimum dose encompassing ≥98% of the GTV, considerably varies and differs between institutions even in the same PD for the same volume BM [12]. Thus, the differences in target definition and dose gradient near the GTV boundary have posed a challenge in developing a consensus regarding the optimal dose for SRS [7,11,12]. Moreover, symptomatic brain radionecrosis (SBR) significantly increases when the surrounding brain volume receiving ≥12 Gy (V_12 Gy_) exceeds >5-10 cm^3^ [13,14]. Therefore, to constrain the V_12 Gy_ <5 cm^3^, the indication of sfSRS has been limited to smaller tumors ≤15 mm or ≤1 cm^3^ [7,14]. In addition, the location of the BM correlates with the risk of adverse radiation effects (ARE) including SBR [8,15]. BM with a deep location of <5 mm from the brain surface (Location grade 2) is susceptible to higher doses of exposure to the surrounding brain when compared to superficially located BM [15]. Additionally, the deep white matter is less likely to tolerate SRS than the cerebral cortex [8,15]. Thus, simply reporting the PD with % IDS does not give the whole picture of the treatment contents [8,11,12]. That specification is also insufficient to objectively compare the different SRS plans for the same case to estimate the efficacy and safety [8,12]. Hence, more detailed and in-depth evaluations and analyses are necessary to determine the optimal dose and distribution [8,12,16]. 

Here, we describe a case of an elderly patient with synchronous, symptomatic, single 20-mm BM located in a deep, eloquent region, originating from LAC, who was treated using sfSRS. Despite the high V_12 Gy_ of >10 cm^3^ and the unintentionally low marginal dose of ≤18 Gy, local complete remission (CR) with minimal ARE was achieved at six months after the SRS and sustained at nearly five years. We will discuss the reasons for the long-term excellent tumor response and being immune to late ARE.

This report was part of the clinical study approved by the Clinical Research Review Board of Kainan Hospital Aichi Prefectural Welfare Federation of Agricultural Cooperatives (20220727-1).

## Case presentation

**Figure 1:**
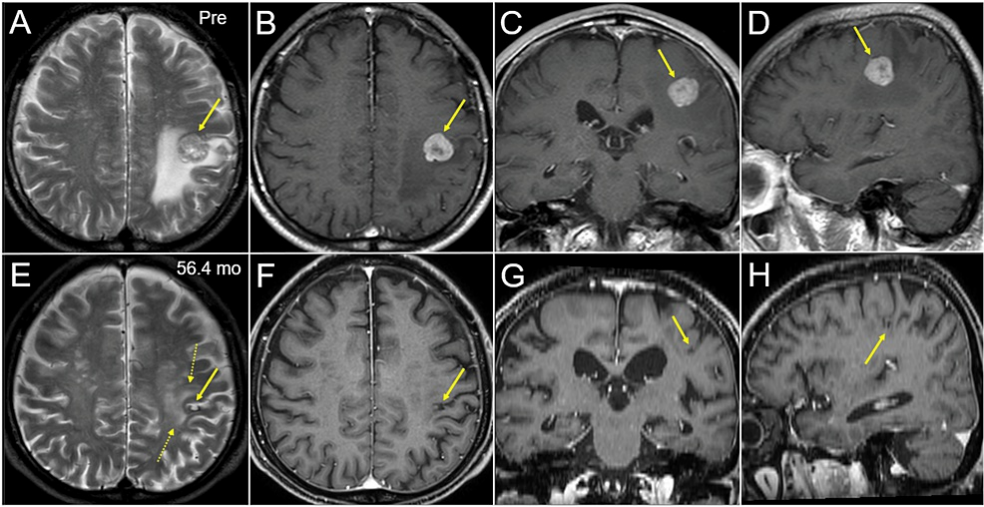
Magnetic resonance images before and 4.7 years after stereotactic radiosurgery. The images show T2-weighted images (WI) (A, E); contrast-enhanced (CE) T1-WI (B-D, F-H); axial images (A, B, E, F); coronal images (C, G); sagittal images (D, H); before stereotactic radiosurgery (SRS) (Pre) (A-D); and at 56.4 months (mo) after SRS (E-H). (A-H) All images were co-registered on MIM Maestro^TM^ software (MIM Software Inc., Beachwood, Ohio, United States) and are shown in the same magnification and coordinates; (A-D) A solid mass lesion (arrows in A-D) in the left frontoparietal lobes is concomitant with surrounding edema almost not extending into the ventral lobe; (E-H) The tumor remnant is observed as a cavitary lesion (arrows in E-H) in the left postcentral gyrus; (E) A slight high-intensity change is seen in the surrounding white matter (dashed arrows in E); (E-H) Progression of brain parenchyma atrophy and relevant ventricular dilatation are seen.

A 75-year-old male experienced jerkiness and weakness of the right hand five months before the diagnosis of synchronous BM from LAC. The patient was an ex-smoker with unremarkable previous medical histories. Magnetic resonance images showed a solid mass lesion with 20 mm in the maximum diameter in the left frontoparietal lobes (Figure 1 A-D).

**Figure 2:**
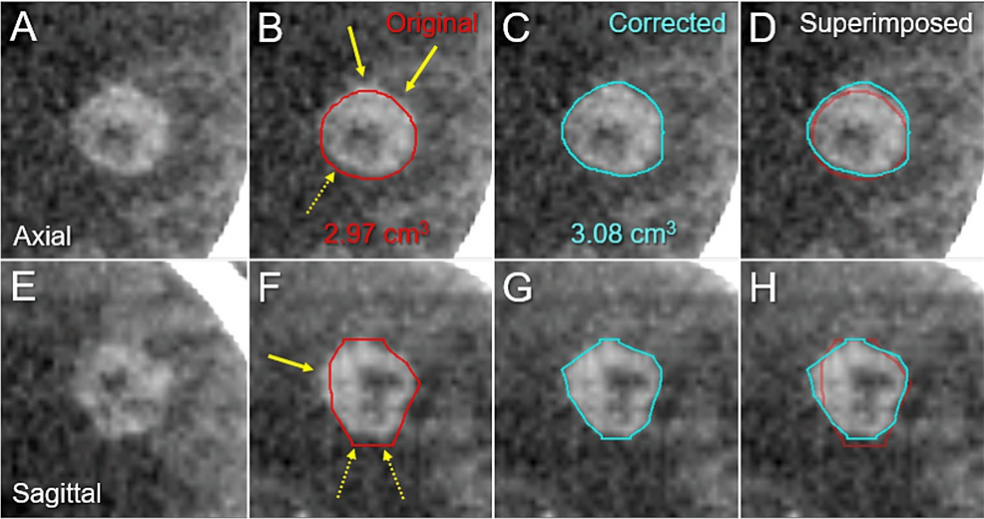
Contrast-enhanced computed tomographic images for stereotactic radiosurgery planning. The images show CECT images (A-H); axial images (A-D); sagittal images (E-H); original contours of the GTV (B, F); corrected contours of the GTV (C, G); and the superimposed images (D, H). (B-D, F-H) The original GTV contours had some under-coverage (arrows in B, F) and over-coverage (dashed arrows in B, F). CECT: contrast-enhanced computed tomography; GTV: gross tumor volume

Biopsy results proved LAC harboring epidermal growth factor receptor (EGFR) L858R mutation in exon 21. ^18^F-fluorodeoxyglucose positron emission tomography(PET)/computed tomography (CT) images revealed that further synchronous metastases were on the right adrenal gland and the fourth lumbar vertebra, indicating oligometastases. The clinical stage was IVB (cT1c N0 M1c) based on the tumor, node, and metastasis (TNM) grading system defined by the eighth edition of the Union for International Cancer Control criteria. The Lung-molGPA score was 2.5, with the expected MST being 26.5 months [4]. sfSRS for BM was commenced by the predecessor radiation oncologist before systemic treatment. The linac system was Infinity® (Elekta AB) with a flattening filter (FF)-free mode of a 6-megavoltage X-ray beam. Volumetric-modulated arc therapy (VMAT) with a 5-mm leaf-width multileaf collimator Agility® (Elekta AB) and a planning system Monaco® (Elekta AB) were used for the optimization of the SRS planning [12]. The GTV (2.97 cm^3^) margin was contoured based on contrast-enhanced (CE) CT images alone with a 1-mm slice thickness (Figure 2). sfSRS was implemented 11 days after the planning CT acquisition, in a frameless manner under image guidance with the head immobilized with a thermoplastic mask.

The PD was a single fraction of 20 Gy that was assigned to the GTV D_95%_. Retrospective review of the GTV definition revealed that the original GTV (oGTV) had some under- and over-coverage of the enhancing lesion (Figure 2). Therefore, the planning parameters were re-evaluated using both the oGTV and the corrected GTV (cGTV) of 3.08 cm^3^ that was contoured under the simultaneous reference of co-registered image dataset including CECT for planning, CE-T1-weighted images (T1-WI), and T2-weighted images (T2-WI) for initial surveillance, using a dedicated software MIM Maestro^TM^ (MIM Software Inc.) (Figure 2) [8,16]. The configurations of the enhancing lesion on CECT and CE-T1-WI as well as the visible mass on T2-WI were almost identical without excessive exudation of contrast media [8,16] (Figure 1).

**Figure 3:**
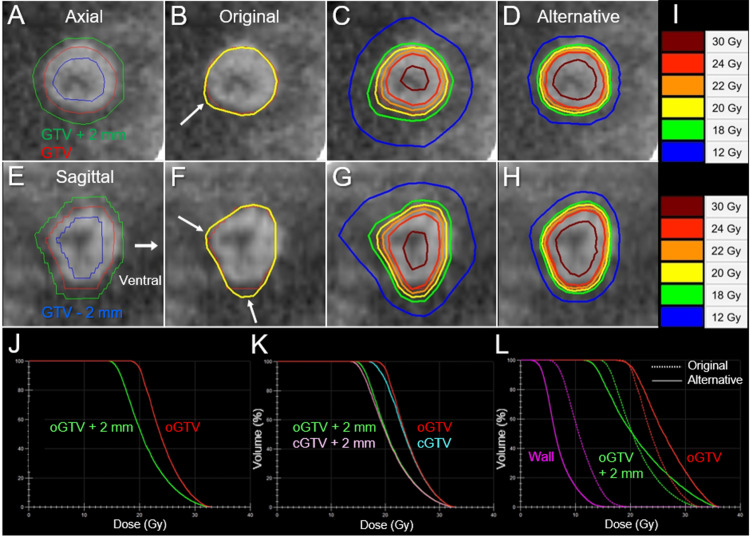
Target definition, dose distributions, and dose-volume histograms. The images show CECT images for SRS planning (A-H); target definition (A, E); dose distributions for the original (B, C, F, G) and alternative plans (D, H); axial images (A-D); sagittal images (arrow in E indicating the ventral side) (E-H); representative isodoses (I); and dose-volume histograms (DVHs) (J-L). (A, E) The original GTV and uniform 2-mm added and reduced objects (GTV + 2 mm, GTV – 2 mm). The contours of margin-added or reduced structures generated on Monaco® (Elekta AB, Stockholm, Sweden) are not smooth but slightly jerky. (B, F) The 20 Gy isodose lines covers 95% of the original GTV (oGTV) with some over-coverage (arrows in B, F). (D, H) The dose gradients just outside and inside the oGTV boundary in the alternative plan (D, H) are steeper and more concentrically laminated than those for the original plan (C, G). (J) The DVHs for the oGTV and oGTV + 2 mm. (K) The DVHs for the corrected GTV (cGTV) and cGTV + 2 mm in addition to the oGTV and oGTV + 2 mm. (L) Comparison of the DVHs for the original and alternative plan. The wall structure is the 8-mm thickness object outside the oGTV + 2 mm boundary. CECT: contrast-enhanced computed tomography; SRS: stereotactic radiosurgery; GTV: gross tumor volume

An alternative plan as a reference was also generated for the oGTV using simpler optimization with an affirmative allowance of increased GTV dose inhomogeneity to enhance the dose conformity and to minimize the surrounding brain dose [11,12], in which the oGTV D_98%_ was rescaled to 19.4 Gy to coincide with that of the original plan. The oGTV, relevant structures for evaluation, dose distributions for the original and alternative plans, and the dosimetric parameters are shown in Figure 3 and Tables 1-2.

**Table 1:** Dosimetric parameters of the original and corrected gross tumor volumes and relevant structures. GTV: gross tumor volume; oGTV: original GTV; cGTV: corrected GTV; D_max_: maximum dose; D_X%_: a minimum dose encompassing X% of the object volume; D_min_: minimum dose

	oGTV	cGTV
D_max_ (D_0.001 cc_)	33.0 Gy	33.0 Gy
GTV D_2%_	31.3 Gy	31.3 Gy
GTV D_50%_	23.8 Gy	23.6 Gy
GTV – 2 mm D_98%_	24.2 Gy	23.2 Gy
GTV – 1 mm D_98%_	22.3 Gy	21.0 Gy
GTV D_95%_	20.0 Gy	18.7 Gy
GTV D_98%_	19.4 Gy	18.0 Gy
GTV D_min_	18.0 Gy	16.1 Gy
GTV + 1 mm D_98%_	17.3 Gy	16.2 Gy
GTV + 2 mm D_98%_	15.4 Gy	14.8 Gy

**Table 2:** Dosimetric parameters of the gross tumor volume and surrounding structures. The irradiated isodose volume is a total volume, including GTV, receiving a specified dose. GTV: gross tumor volume; oGTV: original GTV; cGTV: corrected GTV

Dose	Coverage		Irradiated isodose volume	
	oGTV	cGTV	Original plan	Alternative plan
24 Gy	47.1%	45.3%	1.39 cm^3^	1.96 cm^3^
22 Gy	72.3%	68.1%	2.18 cm^3^	2.49 cm^3^
20 Gy	95.0%	87.3%	3.25 cm^3^	3.12 cm^3^
19.4 Gy	98.0%	91.4%	3.64 cm^3^	3.31 cm^3^
18 Gy	100%	98.1%	4.76 cm^3^	3.82 cm^3^
16 Gy	-	100%	6.93 cm^3^	4.73 cm^3^
14 Gy	-	-	10.01 cm^3^	5.85 cm^3^
12 Gy	-	-	14.32 cm^3^	7.32 cm^3^
10 Gy	-	-	20.13 cm^3^	9.42 cm^3^

In the original plan, the dose spillage volume of 19.4 Gy (oGTV D_98%_) outside the oGTV was 0.93 cm^3^ with an over-coverage of 25.1%. The D_98%_ values of the cGTV and 2-mm outside the cGTV boundary were 18.0 Gy with 55% isodose relative to the maximum dose and 14.8 Gy, respectively. The V_12 Gy_ was >11 cm^3^. In the alternative plan, the V12 Gy decreased to ≤4.24 cm^3^ and the over-coverage of the oGTV with 19.4 Gy decreased to 13.7%. A summary of anti-cancer treatments following sfSRS is demonstrated in Table 3.

**Table 3:** Summary of anti-cancer treatments after initial stereotactic radiosurgery. mo: month; L4: 4^th^ lumbar vertebra; EGFR: epidermal growth factor receptor; CBDCA: carboplatin; PEM: pemetrexed; yo: year old; SRS: stereotactic radiosurgery; BMs: brain metastases

Time	Anti-cancer treatment
0.4 mo	1st line: erlotinib (150 → 50 → 100 → 150 mg/day)
12.0 mo	Palliative radiotherapy for painful L4 metastasis
22.1 mo	Re-biopsy: any EGFR mutation undetected
23.9 mo (77 yo)	2nd line: CBDCA + PEM
39.4 mo	2nd SRS for two new BMs
39.8 mo (79 yo)	3rd line: S-1
45.0 mo	Discontinuation of anti-cancer medication
48.7 mo	3rd SRS for three new BMs
53.8 mo	4th SRS for two new BMs
56.4 mo	Progression of the primary site and thoracic lymph nodes
60.0 mo (80 yo)	Deceased

**Figure 4:**
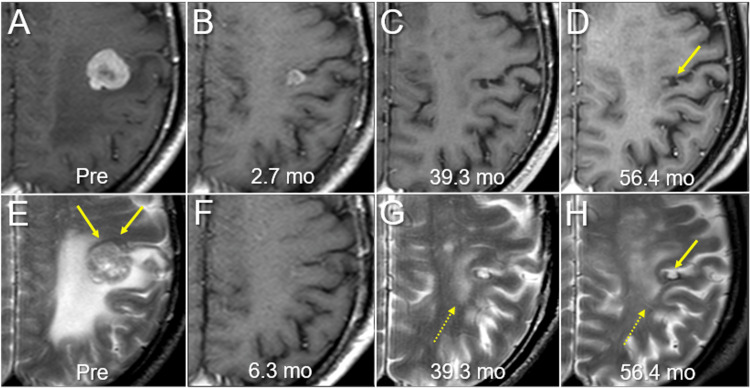
Magnetic resonance images before and after radiosurgery for brain metastasis. The images show axial CE-T1-WI (A-D, F); axial T2-WI (E, G, H); 12 days before SRS (Pre) (A, E); at 2.7 months (mo) after SRS (B); at 6.3 months (F); at 39.3 months (C, G); and at 56.4 months (D, H). (B, F) T2-WI at 2.7 and 6.3 months after SRS were not acquired. All images were co-registered and are shown in the same magnification and coordinates. (E) The iso-intensity brain structure (arrows in F), probably the cerebral cortex, is distinguishable from the BM with heterogeneous high intensity. (B) Remarkable tumor shrinkage along with almost disappearance of the peritumoral edema is observed at 2.7 months. (F) Almost complete remission is confirmed at 6.3 months. (C, D, G, H) At 39.3 and 56.4 months, the tumor remnant (arrows in D, H) is visible as a cavitary lesion surrounded by a low-intensity rim on T2-WI with not the whole circumference, just bordering the central sulcus at the ventral side. No abnormal intensity is observed in the surrounding iso-intensity cerebral cortex, although high-intensity changes are observed in the surrounding deep white matter (dashed arrows in G, H). CE: contrast-enhanced; WI: weighted image; SRS: stereotactic radiosurgery; BM: brain metastasis

The patient's right-sided weakness increased 10 days after SRS; however, he eventually recovered fully. Erlotinib was administered at a dose of 150 mg/day 13 days after SRS and was reduced further to 50 mg/day owing to liver dysfunction 26 days after the initiation. Remarkable tumor shrinkage and subsequent nearly CR were confirmed at 2.7 and 6.3 months, respectively, while at 56.4 months, the tumor remnant was visible as a tiny cavitary lesion that was mainly located in the cerebral cortex of the left post-central gyrus (Figures 1, 4).

Post-SRS intracranial progression was limited to a total of seven new BMs that were successfully salvaged by three additional SRS at 39.4, 48.7, and 53.8 months, respectively, without whole brain irradiation (Table 3). The patient died 60.0 months after the first SRS, mainly owing to intrathoracic progression. A head CT 16 days before his death showed no evidence of progression and/or ARE of the initial BM (data not shown).

## Discussion

In the updated analysis of the JLGK0901 study, the cumulative local tumor progression (LTP) showed 17.8% in the entire cohort, including 1194 cases of BM <10 cm^3^ or <3 cm treated with sfSRS using GK, which was defined as a ≥20% increase in the maximum diameter from the nadir response [9]. The pathological conditions of LTP can include tumor regrowth following partial response, ARE including BR, and the mixed condition. The significant contributing factors for LTP included GTV ≥1 cm^3^ and PD of <22 Gy [9]. In addition, the cumulative incidence of post-sfSRS adverse events of grade ≥2 reached 12.6% of the BM cases from non-small cell lung cancer (NSCLC) [17]. This suggests that the indication of sfSRS should be limited to smaller tumors of <1.5-2 cm to enhance safety [7,12,14]. In multi-fraction (mf) SRS with 2-10 fractions (fr), the linear-quadratic model-derived biological effective dose with the alpha/beta ratio of 10 (BED_10_) of ≥80 Gy, the equivalent single dose of ≥23.8 Gy, provides better LTC for BMs from NSCLC [18]. Improving conventional treatment outcomes for both efficacy and safety, our principles of mfSRS for BM since 2018 have been BED_10_-based three-tiered dose preservation with a various and flexible number of fractionations from 3-15 fr: ≈50 Gy for 2-3 mm outside the GTV boundary, ≥80 Gy for the GTV margin, and ≥100 Gy for 2 mm inside the GTV boundary [8,12,16]. For a 2-cm BM with a deep, eloquent location, similar to the present case, ≥43 Gy in ≥5 fr is usually assigned to ≥98% of the GTV [12].

In the present case, by employing a combination of sfSRS followed by erlotinib, a sustained CR lasting nearly five years was achieved with minimal ARE, which suggests that from an outcome-centric perspective, the SRS method was one of the most suitable treatment contents for this patient. However, the CE-CT-based GTV delineation without CE-T1-WI and T2-WI matching can underestimate the accurate boundary of the GTV. Furthermore, the coverage of the cGTV with the intended PD of 20 Gy was 87.3%, meaning that a tumor volume of 0.39 cm^3^ (12.7% of 3.08 cm^3^), equivalent to a 9-mm sphere-shaped tumor, was <20 Gy. Although the planned cGTV D_98%_ was 18 Gy, the actual D_98%_ could have been lower, considering potential enlargement and/or altered edema-related displacement of the GTV during the 11 days after planning CT acquisition [19]. Despite these potential shortcomings and flaws, the remarkable tumor response with long-term sustainment suggests the presence of radio-sensitive and TKI-sensitive LAC-BM, for which sfSRS with GTV marginal dose ≤18 Gy combined with TKI is sufficient for tumor eradication. In mfSRS with synergistic use of EGFR-TKI, the GTV marginal dose with the BED_10_ of ≤50-70 Gy may be adequate for some LAC-BMs.

The original plan exhibited substantial scope for improvement for the dose conformity; the dose fall-off outside the GTV was gradual, which inevitably increased the dose exposed to the surrounding brain [11,12]. In fact, this patient experienced transient worsening of neurological symptoms after SRS. The different VMAT optimization enabled a significant reduction of the high V_12 Gy_ by 62% (Figure 3, Table 2). Despite the high V_12 Gy_, however, the late ARE was minimal. The main involvement of the BM in the cerebral cortex likely rendered the present case less susceptible to ARE compared to a location in the deep white matter. In addition, the total volume receiving ≥22 Gy (22 Gy volume) was 2.18 cm^3^ (<GTV), although the 22 Gy volume of ≥2.62 cm^3^ significantly increases the risk of SBR [15]. We previously reported that the balance between the high (22 Gy) and low (12 Gy) dose-irradiated volumes can predict the risk of ARE more accurately than the low dose volume alone [15]. Additionally, the moderate GTV marginal dose would also contribute to the avoidance of ARE. Thus, the risk of late ARE relevant to sfSRS would be predicted more accurately by comprehensive estimation including the relation of BM location with the cerebral cortex and high dose irradiated volume outside the GTV, in addition to V12 Gy. Furthermore, the maximum response of CR with complete extrication from the existing mass effect may also render the surrounding brain tissue less susceptible to late ARE. Meanwhile, the GTV over-coverage and a rather gradual dose gradient outside the GTV can cover the inherent inaccuracies of irradiation, including the potential enlargement during the interval between planning image acquisition and SRS initiation, microscopic brain invasion, and intra-fractional movement [7,19,20]. Additionally, although the cGTV was covered with a minimum dose of 16.1 Gy with 48.8% isodose similar to LGK [9], the long-term local treatment outcome was excellent. This also suggests that an extremely heterogeneous GTV dose would not be detrimental for therapeutic efficacy and/or safety [12].

The intrinsic radiosensitivity, pharmacosensitivity, and the environment within the tumor (oxygenation state) and at the brain-tumor interface, including microscopic brain invasion, for LAC-BM would appear to be substantially heterogeneous. Thus, the minimum required dose for achieving CR would vary and differ between individual cases [7,12,20]. Considering the current situation where it is difficult to predict the level of radiosensitivity in advance, it is preferable to ensure adequate dosage while adopting a flexible and suitable number of fractionation to attain excellent tumor response with high probability along with minimum ARE [8,12]. The present case warrants further investigation identifying the characteristic features or biomarkers of a subset of LAC-BM cases, for which SRS dosage can be reduced without compromising on excellent anti-tumor efficacy.

## Conclusions

The present case showed existence of extremely radio- and TKI-sensitive LAC-BM for which single fraction of ≤18 Gy combined with TKI is sufficient for attaining long-term CR and long-term brain tolerance following sfSRS despite the high 12 Gy volume and deep eloquent location in the late 70s.
